# Efficacy of Ophthalmic Viscosurgical Device–Assisted Vitrectomy for Membrane Removal in Patients With Complex Proliferative Diabetic Retinopathy

**DOI:** 10.1155/joph/5436368

**Published:** 2026-03-01

**Authors:** Su Zhang, Hui-Ying Zhang, Lushu Chen, Qin Jiang, Jin Yao

**Affiliations:** ^1^ Department of Ophthalmology, The Affiliated Eye Hospital of Nanjing Medical University, Nanjing, China, jseye.com.cn

**Keywords:** complex proliferative diabetic retinopathy, ophthalmic viscosurgical device, vitrectomy

## Abstract

**Aim:**

To assess the application of an ophthalmic viscosurgical device (OVD) for stripping proliferative membranes during vitrectomy in patients with complex proliferative diabetic retinopathy (PDR).

**Methods:**

This was a prospective cohort study that enrolled 28 patients (30 eyes) diagnosed with complex PDR at the Affiliated Eye Hospital of Nanjing Medical University. The patients were randomly divided into two groups based on different surgical procedures: The patients who received vitrectomy combined with OVD‐assisted internal limiting membrane peeling were assigned to Group A (15 eyes of 13 patients), while Group B patients (15 eyes of 15 patients) underwent vitrectomy without OVD‐assisted internal limiting membrane peeling. The primary outcomes measured included intraoperative bleeding, operation duration, electrocoagulation for hemostasis, occurrence of iatrogenic retinal holes, postoperative best‐corrected visual acuity (BCVA), and complications such as recurrent retinal detachment and vitreous hemorrhage.

**Results:**

Compared with the mean preoperative BCVA, the mean postoperative BCVA at the last follow‐up improved significantly in both groups (all *p* < 0.05), but no significant difference in mean BCVA improvement was found between the two groups (*p* > 0.05). Regarding intraoperative complications, no iatrogenic retinal tears or retinal detachments occurred in any group. Intraoperative observations revealed that the preretinal injection of OVD facilitated the rapid separation of fibrous vascular membranes from the retina, while also serving a hemostatic function, thus maintaining a clear visual field. In Group A, photocoagulation was utilized for hemostasis in two eyes, whereas in Group B, it was employed in 9 cases, with a statistically significant difference between the groups (*p* < 0.05). The average surgical duration was 37.47 ± 4.69 min for Group A and 52 ± 6.26 min for Group B, with a statistically significant difference noted (*p* < 0.05). No iatrogenic retinal holes, recurrent vitreous hemorrhages, retinal detachments, choroidal hemorrhages, or endophthalmitis were observed in both groups.

**Conclusion:**

Our study demonstrates a new application of OVD in proliferative membrane removal during vitrectomy, particularly in patients with PDR characterized by strong adhesions and complex structures. This method offers a potentially safer and more effective option for treating patients with PDR.

## 1. Introduction

Diabetic retinopathy (DR) is one of the most common microvascular complications of diabetes and is the leading cause of blindness among working‐age adults in numerous countries [1]. Proliferative diabetic retinopathy (PDR), a prevalent and severe form of DR, affects approximately 1.4% of all individuals with diabetes [2] and significantly threatens vision. The hallmark of PDR is retinal neovascularization, which can lead to vitreous hemorrhage due to leakage from unstable blood vessels. Fibrous hyperplasia, another critical characteristic of PDR, results in progressive fibrosis and tractional retinal detachment, which ultimately impairs vision and causes blindness.

Currently, treatment options for PDR include laser photocoagulation, antivascular endothelial growth factor (anti‐VEGF) therapy, and if necessary, pars plana vitrectomy (PPV) [3]. PPV is an effective clinical intervention for patients with persistent or recurrent severe vitreous hemorrhage, neovascular membrane formation, and tractional retinal detachment. This procedure enhances visual function by removing the vitreous cavity hemorrhage and fibrous tissue [4]. PPV involves using a cutter or microintraocular scissors to carefully detach the fibrovascular membrane from the retina at the anterior‐retinal interface. However, in cases of tightly adherent and complexly structured membranes that challenge precise delineation, relying solely on conventional tools can increase the risk of intraoperative bleeding, postoperative recurrent vitreous hemorrhage from vascular damage, and iatrogenic retinal tears from excessive retinal trauma [5]. Ophthalmic viscosurgical device (OVD) is a specialized, gel‐like material with unique physical properties that facilitates space creation and protection in the vitreoretinal surgical field, aid in the separation of closely adhered complex tissues [6], and provide hemostatic benefits [7]. As early as 1983, Stenkula and Toquist applied sodium hyaluronate to dissect epiretinal fibrovascular and fibroglial membranes by raising and separating them from the retina’s surface in eyes with severe PDR [8]. However, by 1988, McLeod and James demonstrated that sodium hyaluronate was of great value in elevating vitreous cortex or sparsely vascularized epiretinal membranes, especially in eyes with combined traction and rhegmatogenous retinal detachment. However, bleeding from or tearing of the retina limited the usefulness of this technique in the surgery of highly vascularized and adherent membranes, as in eyes with table‐top traction retinal detachment. Recurrent epiretinal membrane proliferation was seen in some eyes postoperatively. They concluded that the shortfalls of this technique would restrict its use when compared to strictly mechanical procedures [9]. It is therefore evident that further research is warranted to confirm the clinical efficacy of OVDs for PDR surgeries. This study retrospectively evaluated the safety and efficacy of OVD‐assisted proliferative membrane removal in patients with PDR undergoing PPV, aiming to provide a safer and more effective treatment alternative.

## 2. Materials and Methods

### 2.1. Study Participants

The study included 28 patients (30 eyes) diagnosed with complex PDR, all of whom underwent vitrectomy. Patients with complex PDR were diagnosed according to the Diabetic Retinopathy Preferred Practice Pattern Guidelines issued by the American Academy of Ophthalmology and were classified as having high‐risk PDR. Surgical intervention is indicated for patients presenting with proliferative fibrovascular membranes. All patients diagnosed with high‐risk PDR received intravitreal anti‐VEGF injections within 3–5 days before surgery. The patients were randomly assigned to two separate groups according to the surgical methods used. Group A, comprising 15 eyes from 13 patients, underwent vitrectomy along with OVD‐assisted proliferative membrane removal. In comparison, Group B, which consisted of 15 eyes from 15 patients, underwent vitrectomy only, without the additional viscoelastic‐assisted peeling step. The exclusion criteria were as follows: (1) absence of light perception in the eye prior to surgery; (2) history of vitrectomy; (3) previous eye conditions affecting vision, such as the macular hole, uveitis, primary glaucoma, ocular tumor, or ocular trauma; and (4) preoperative iris or angle neovascularization or elevated intraocular pressure.

### 2.2. Ethics Approval and Consent to Participate

This study was designed and performed in accordance with the Declaration of Helsinki and approved by the Ethics Committee of the Affiliated Eye Hospital of Nanjing Medical University (2022006). All participants provided written informed consent.

### 2.3. Surgical Technique

All procedures were performed by the same surgeon. A minimally invasive 25‐gauge pars plana vitrectomy (25G PPV) was performed under general anesthesia. This procedure involved creating a 25G scleral incision 3.5 mm behind the corneoscleral limbus in the infratemporal quadrant for optic fiber illumination and vitrectomy using equipment from Alcon (USA). In certain instances, an additional 25G scleral incision was made directly above, and a ceiling lamp lighting system was employed to facilitate membrane peeling or bleeding control when both hands were required (Alcon, USA) (Figure 1(A)). Phacoemulsification for cataract extraction was performed in patients with significant lens opacity. A noncontact wide‐angle observation system (Resight 700, Carl Zeiss Meditec AG, Germany) enabled a thorough and clear fundus examination, followed by vitreous hemorrhage removal to induce posterior vitreous detachment (PVD). The fibrovascular membrane was carefully segmented, layered, and peeled. For membranes that were tightly adhered or complex, making accurate distinction challenging, a special injection needle (Figure 1(B)) was used to precisely inject the OVD (Healon®, Johnson & Johnson Vision, USA) with high viscosity between the proliferative membrane and the retina. A video recording of the dynamic removal of the proliferative membrane assisted by viscoelastic injection during PPV is provided in Supporting Video S1. This technique facilitated the safe separation of the membrane, minimizing the risk of retinal breaks during peeling. When intraoperative bleeding occurred, OVD was applied to manage the active bleeding. Uncontrollable bleeding necessitated electrocoagulation to achieve hemostasis. Comprehensive pan‐retinal photocoagulation was applied to all treated eyes. Eyes with retinal detachment were treated with silicone oil.

FIGURE 1Schematic diagram of PPV surgery. (a) Microscopic view. (b) Schematic diagram of the needle used for the ophthalmic viscosurgical device. (1) The tailstock of the needle, which is equipped with a centrally symmetric connecting plate resembling a propeller blade for easy rotation, is connected to the ophthalmic viscosurgical device. (2) A needle tube measuring 40 mm in length and 25G in diameter was used.

**Figure:**
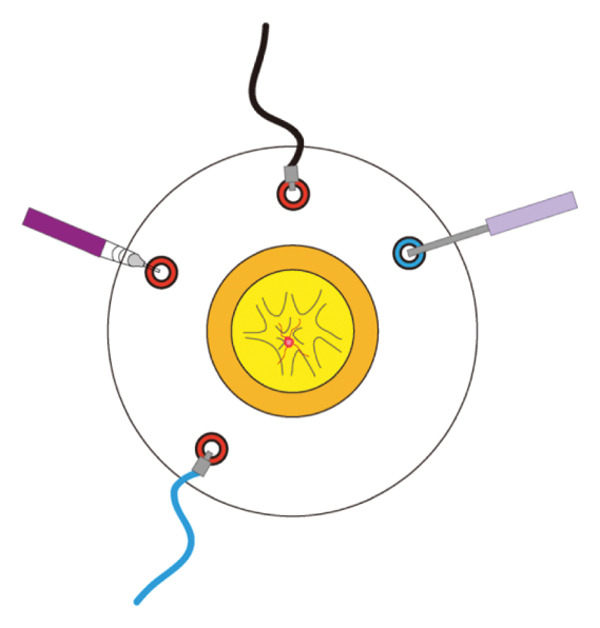
(a)

**Figure:**
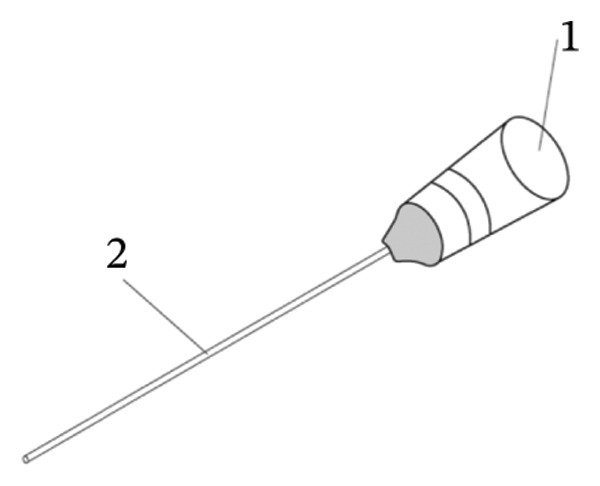
(b)

### 2.4. Main Outcome Measures

Key intraoperative metrics including bleeding, operation duration, instances of electrocoagulation hemostasis, and iatrogenic retinal breaks were meticulously documented. Postoperative visual acuity, intraocular pressure, wide‐angle fundus photography, and optical coherence tomography (OCT) were routinely assessed. This study reported a follow‐up duration of less than 1 month.

### 2.5. Statistical Analysis

All statistical analyses were performed using GraphPad Prism 9 software (GraphPad Software, USA). Data are presented as the mean ± standard deviation (SD), and categorical variables are presented as frequencies and percentages. Paired *t*‐test was used to compare preoperative and postoperative continuous variables. Statistical significance was set at *p* value < 0.05.

## 3. Results

### 3.1. Baseline Characteristic of Study Population

In this research, both groups of patients successfully completed the operation. Demographic and clinical details of the subjects are presented in Table 1.

**TABLE 1:** Baseline characteristics of study population.

	Group A	Group B	*p* value
Age (years)	51.15 ± 8.04	54.73 ± 9.7	0.3021
Sex (male/female)	6/7	9/6	0.7539
IOP (mmHg)	14 ± 2.27	14.8 ± 2.83	0.4005
BCVA (LogMAR)			
Preoperative	1.87 ± 0.82	1.56 ± 0.95	0.3401
Postoperative	1.11 ± 0.57^∗∗^	0.83 ± 0.44^∗^	0.1443
Δ	0.77 ± 0.74	0.73 ± 0.9	0.9123

^∗^ *p* < 0.05.

^∗∗^ *p* < 0.01, Preoperative BCVA (LogMAR) versus postoperative BCVA (LogMAR).

### 3.2. Intraoperative Status and Complications

The intraoperative conditions and complications of the two groups of patients are summarized in Table 2. The average operation time was 37.47 ± 4.69 min in Group A and 52 ± 6.26 min in Group B, with a statistically significant difference noted (*p* < 0.05). Intraoperative findings indicated reduced bleeding when the OVD assisted in membrane removal. Notably, six eyes exhibited significant bleeding during membrane excision, with most instances of bleeding ceasing following OVD application. In two patients, active bleeding persisted despite viscoelastic compression, necessitating electrocoagulation for hemostasis. Electrocoagulation was employed in 9 cases in Group B, and the intergroup difference was statistically significant (*p* < 0.05). No iatrogenic retinal holes were identified in either study group.

**TABLE 2:** Intraoperative status and complications.

	Surgery time (min)	Electrocoagulation	Iatrogenic retinal break
A group	37.47 ± 4.69	2/15	0/15
B group	52 ± 6.26	9/15	0/15
*p* value	< 0.0001	0.0067	—

### 3.3. Postoperative Complications

**FIGURE 2:**
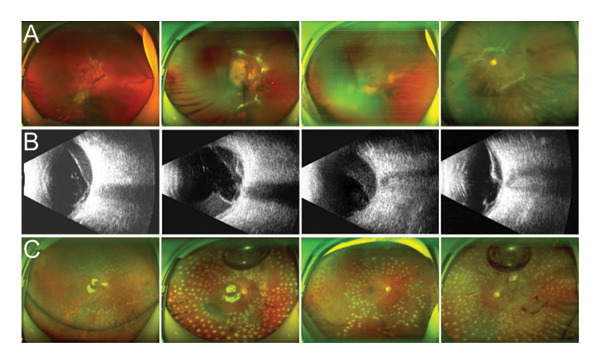
Four cases showed the proliferative membrane was successfully removed by OVD during PPV surgery in Group A. (A) Wide‐angle fundus photographs of 4 patients illustrating the presence of proliferative fibrovascular membranes in the fundus. (B) Preoperative B‐ultrasound examination results for 4 patients, indicating tractional detachment. (C) Wide‐angle fundus photographs of 4 patients 1 week postsurgery, confirming the complete removal of the fibrous vascular membrane.

**FIGURE 3:**
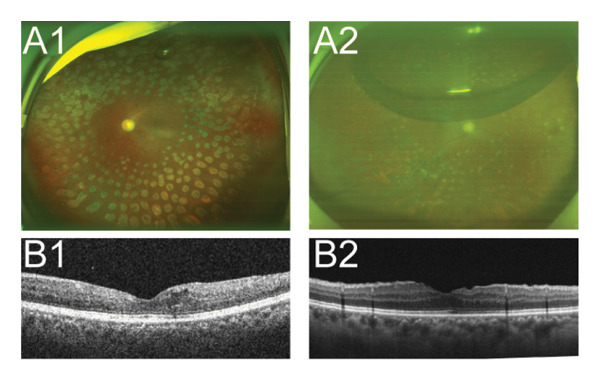
Two cases showed retinal recovery after the removal of silicone oil in Group A. (A1‐2) Wide‐angle fundus photographs of two patients 1 month after silicone oil removal. (B1‐2) OCT images of two patients 1 week after silicone oil removal.

Postoperative assessments, including visual acuity, IOP, wide‐angle fundus photography, and OCT, were conducted. As illustrated in Figure 2, complete removal of the fibrovascular membrane was achieved, with the retina appearing flat and attached, and no active bleeding was noted. The mean postoperative best‐corrected visual acuity (BCVA) (LogMAR) was significantly improved compared with the preoperative value at the last follow‐up visit in both groups, as shown in Table 1. Improvement in vision was greater in Group A than in Group B, but there was no statistical difference between the two groups. Three months later, 11 of the 15 eyes underwent vitrectomy for silicone oil removal, and no new bleeding or retinal detachment was observed (Figure 3). No cases of macular holes, recurrent vitreous hemorrhage, retinal detachment, choroidal hemorrhage, or endophthalmitis were reported.

## 4. Discussion

The OVD is used extensively in intraocular lens implantation, penetrating keratoplasty, and ocular trauma microsurgery [10]. Moreover, these agents are uniquely beneficial for retinal surgery initially serving as vitreous substitutes, using OVD to keep constant intraocular pressure of the eyeball during peeling epiretinal membrane under silicone oil status [11]. Recent studies have expanded their applications to vitrectomy. Previous research has underscored the significant role of the OVD in the repair of macular holes [12]. Muni et al. showed that suprachoroidal viscopexy is feasible as an in‐office technique to create a temporary choroidal buckle for RRD repair [13]. In this study, OVD was employed to remove proliferative membranes in patients with PDR, resulting in a notable enhancement in surgical efficiency compared with conventional methods.

Extensive research has demonstrated that a persistently high‐glucose environment can induce molecular and structural alterations in the vitreous of DR patients, leading to disorders in vitreous collagen cross‐linking and hyaluronic acid degradation [14–16]. This environment simultaneously affects the internal limiting membrane, posterior vitreous cortex, and the extracellular matrix between them, resulting in vitreous adhesion and splitting. Changes at the vitreoretinal interface facilitate the proliferation of neovascularization in the vitreous and the formation of anterior membranes [17]. Given the crucial involvement of the vitreoretinal interface in the pathogenesis of DR, it is imperative to incorporate PVD and complete vitrectomy during PPV. This approach aims to minimize potential postoperative complications, including hemorrhage and epiretinal and proliferative membrane formation, in patients with DR. Our clinical experience indicates that achieving a nasal PVD is particularly challenging especially when dense proliferative membranes and retinal adhesions are present nasally, particularly in the upper nasal quadrant. Extensive fibrotic membranes can grow close to the retinal surface, creating a platform‐like traction with minimal space between the organized tissue and the retina, which is the most challenging aspect of vitrectomy for DR patients. With advancements in cutting technology, skilled surgeons have adopted shovel‐cutting techniques in conjunction with high‐speed, minimally invasive cutting systems [18]. They use a beveled cutting head to excavate the potential gap between the proliferative membrane and the underlying retina, allowing plaque tissue to be gradually suctioned into the cutting head for removal while controlling the excision of residual tissue at a low flow rate. Manual membrane stripping is employed in cases with substantial residual tissue [19]. However, in cases with dense proliferative membranes, tight adhesion to the retina, or preexisting retinal detachment, the entire retina can become edematous. In such cases, the proliferative membrane is indistinguishable from the retina, with no discernible gap. Consequently, the procedure becomes exceedingly difficult and prone to complications, such as iatrogenic retinal holes and hemorrhage [20, 21].

In this study, we enhanced the application of OVD during vitrectomy in patients with PDR presenting with retinal neovascularization and/or tractional retinal detachment. A specialized injection needle was employed to deliver the OVD into the narrow space where the proliferative membrane was tightly adhered to the retina. Through the application of the OVD, gentle separation was achieved, enlarging the gap and enhancing the visibility of the retinal blood vessels, thereby preventing vascular damage and retinal holes. In the initial phase, particularly in patients with significant adhesion of the proliferative membrane to the retina or when traction was combined with rhegmatogenous retinal detachment, an additional fiber‐optic lighting system was positioned above the retina for a four‐incision vitrectomy. The procedure was conducted bimanually: One hand delicately elevated the edge of the proliferative membrane in the affected eye temporally, whereas the other hand administered OVD into the created gap. Subsequently, the surgeon used intraocular scissors or a vitrectomy probe, carefully navigated along the membrane, and methodically detached the adhesion between the proliferative membrane and the retina before completely removing the membrane. As proficiency in viscoelastic use advanced, most later procedures reverted to the conventional three‐incision vitrectomy technique. Peeling was performed single‐handedly by introducing the OVD between the proliferative membrane and retinal adhesion to detach the membrane from the retina, thereby expanding the surgical field. Finally, the entire proliferative membrane was meticulously removed using intraocular scissors. In all the reviewed cases, there were no significant differences between the two groups with different surgical approaches in terms of final retinal anatomical reattachment and visual prognosis, which was consistent with the findings of Grigorian et al. [22, 23] and Fortun and Hubbard [24]. No iatrogenic retinal breaks occurred in any of the reviewed cases, which was inconsistent with the results of Grigorian’s study. The possible reasons for this discrepancy may be attributed to the differences in the number of reviewed cases and the complexity of the conditions involved. The findings of Grigorian et al.’s study indicated that iatrogenic retinal breaks were encountered in 18% of eyes in the viscodissection group and in 11% of eyes in the nonviscodissection group, but the difference was not statistically significant. The frequency of iatrogenic retinal breaks in cases done without viscodissection tended to increase with increasing complexity score, whereas among cases done with viscodissection, the frequency of retinal breaks tended to be independent of case complexity [22, 23].

Furthermore, OVD exhibits a notable hemostatic effect [7]. In the cases studied, six eyes experienced significant bleeding upon the removal of the proliferative membrane. Bleeding was effectively managed by applying an OVD, which compressed the bleeding site. This intervention successfully halted most bleeding events and rendered the surgical field clear, thereby reducing the operation time and enhancing the success rate. In two instances, active bleeding persisted despite viscoelastic compression, necessitating the use of electrocoagulation. No cases of active bleeding were reported at the time of surgery. In our study, the average operation time was considerably shorter, likely due to the use of OVD to assist in the stripping of membranes. The use of OVD to manage proliferative membranes significantly improves the efficiency of complex PDR vitrectomies. Additionally, 13 eyes were treated with silicone oil due to severe retinal compromise caused by ischemia, edema, necrosis, and other factors. Our findings demonstrate a significant improvement in the average postoperative BCVA, which is consistent with prior research [25]. No complications, such as macular holes, recurrent vitreous hemorrhage, or retinal detachment, were observed. Of course, we do not consider that OVD assistance is required for all PDR surgeries. Based on our clinical experience, OVD is indicated for two main scenarios: first, ophthalmologists with relatively limited experience in proliferative membrane dissection; second, specific cases including young patients with complex PDR presenting with incomplete or absent PVD, especially those with extensive, densely adherent fibrovascular membranes attached to the midperipheral or equatorial retina, as well as cases of tractional retinal detachment with or without associated retinal breaks.

This study has several limitations, including a small sample size and a relatively short follow‐up period. Further studies are required to validate the long‐term safety and efficacy of this procedure. In conclusion, our study supports the use of OVD‐assisted vitrectomy as an innovative approach for treating DR characterized by neovascular proliferative membranes and tractional retinal detachment, thereby facilitating improved visual function recovery and quality.

## 5. Conclusions

In this study, we demonstrated that the new application of OVD facilitated successful membrane removal during PPV in patients with complex PDR, significantly improved BCVA, and minimized complications. Additionally, the use of OVD to manage proliferative membranes significantly improved the efficiency of PPV. OVD is a safe and effective treatment for removing proliferative membranes during PPV in patients with complex PDR.

## Author Contributions

Hui‐Ying Zhang and Su Zhang contributed equally. Jin Yao: writing–review and editing, conceptualization, funding acquisition, investigation, project administration, and resources. Qin Jiang: investigation, project administration, and resources. Hui‐Ying Zhang and Su Zhang: writing–original draft, writing–review and editing, data curation, formal analysis, investigation, and methodology. Lushu Chen: formal analysis, investigation, and methodology.

## Funding

This work was supported by grants from the National Natural Science Foundation of China (No. 82271107) and the Key Scientific Research Project of Jiangsu Health Commission (No. K2023060).

## Disclosure

All authors have reviewed and approved the manuscript.

## Conflicts of Interest

The authors declare no conflicts of interest.

## Supporting Information

Supporting Video S1: A video recording of the dynamic removal of proliferative membrane assisted by viscoelastic injection during PPV.

## Supporting information


**Supporting Information** Additional supporting information can be found online in the Supporting Information section.

## Data Availability

The datasets used and/or analyzed in the current study are available from the corresponding author upon reasonable request.
